# Supplementary material for: Structural basis for the ARF GAP activity and specificity of the C9orf72 complex
Source: Nat Commun. 2021 Jun 18;12:3786. doi: 10.1038/s41467-021-24081-0 (PMC8213707; doi:10.1038/s41467-021-24081-0)
Supplement: Supplementary file 1 — Supplementary Information [file 41467_2021_24081_MOESM1_ESM.pdf]

Supplementary Information

Structural basis for the ARF GAP activity and specificity of the C9orf72 complex

Ming-Yuan Su[1,2,3], Simon A. Fromm[2,3,4], Jonathan Remis[3], Daniel B. Toso[3], and James H. Hurley[2,3*]

[1]School of Medicine, Southern University of Science and Technology, Shenzhen, 518055, China

[2]Department of Molecular and Cell Biology, University of California, Berkeley, California, USA

[3]California Institute for Quantitative Biosciences, University of California, Berkeley, California, USA

[4]Present address: Imaging Centre, European Molecular Biology Laboratory, Heidelberg, Germany

*corresponding author: jimhurley@berkeley.edu

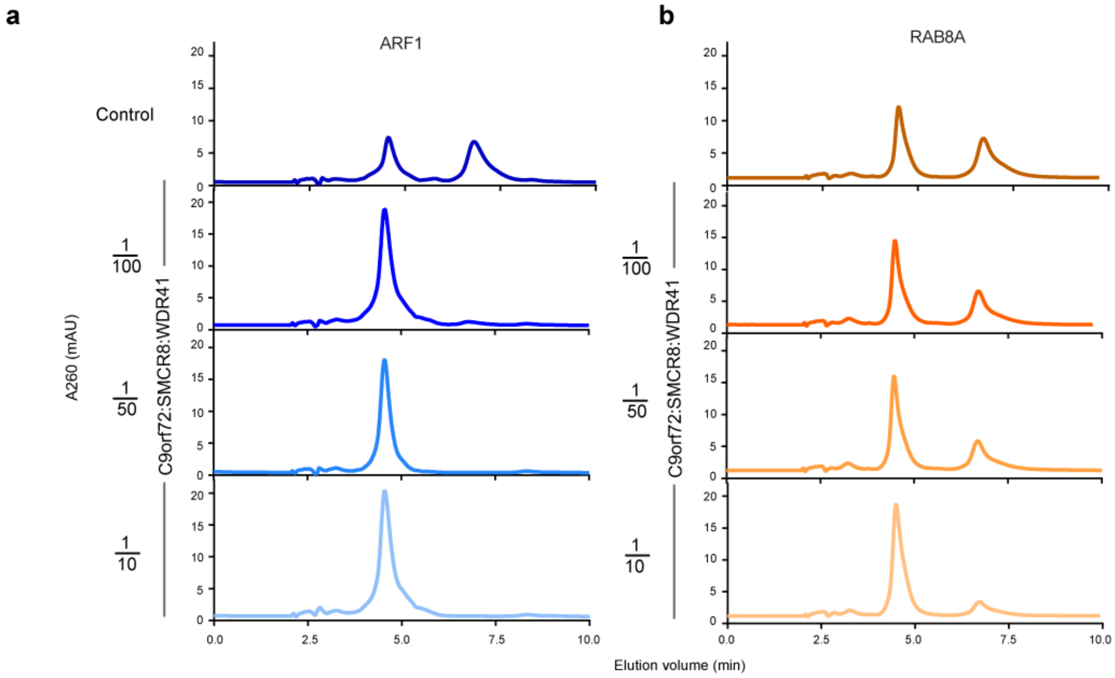

Supplementary Fig 1. The HPLC result of incubating ARF1 and RAB8A with different molar ratio of C9orf72:SMCR8:WDR41 complex.

30 μM of ARF1 (a) or RAB8A (b) was treated with 0, 0.3, 0.6 and 3 μM C9orf72:SMCR8:WDR41 complex for 15 min at 37°C.

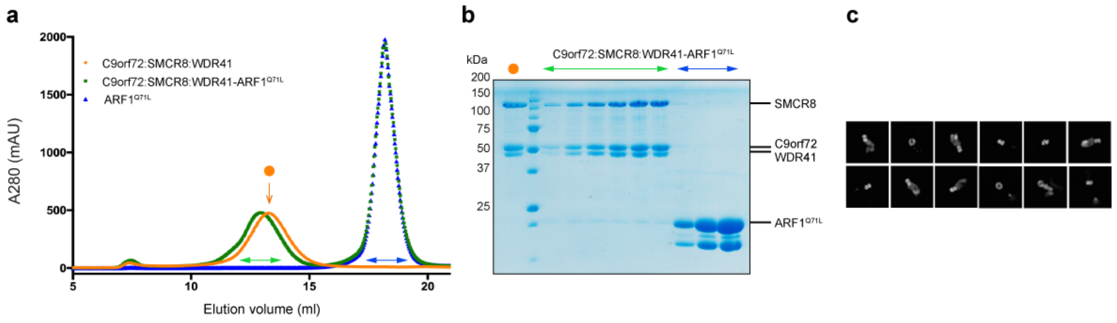

Supplementary Fig 2. Assembly of C9orf72:SMCR8:WDR41:ARF1^{Q71L}.

a, the Superose 6 size exclusion profile of the reconstituted C9orf72:SMCR8:WDR41:ARF1^{Q71L} complex. b, the SDS-PAGE analysis of the peak fractions. For all the lanes, one representative result from at least two independent experiments is shown. c. 2D class averages for the C9orf72:SMCR8:WDR41:ARF1^{Q71L} complex.

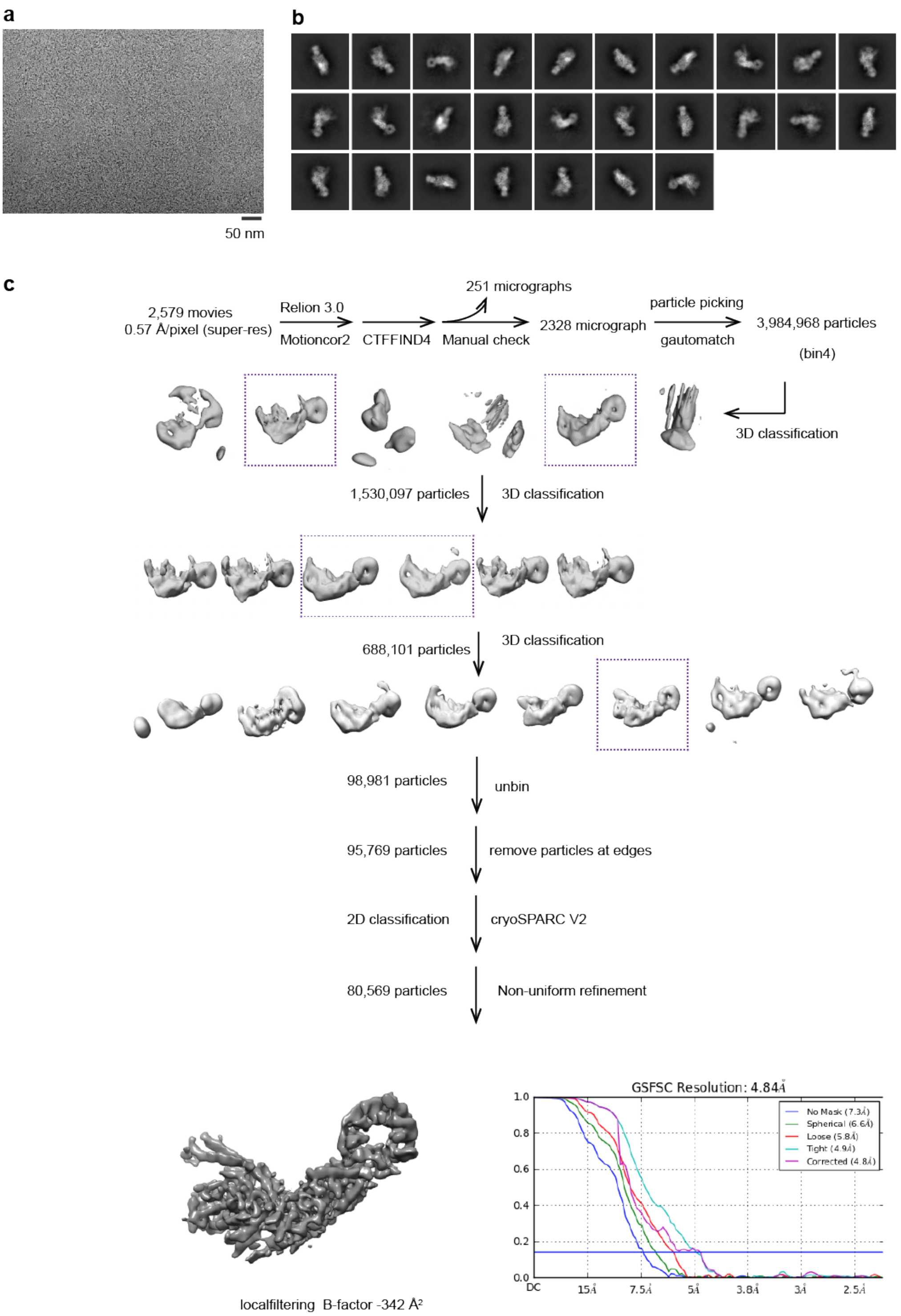

Supplementary Fig 3. Cryo-EM data processing work flow for C9orf72:ARF1-SMCR8:WDR41 complex incubated with BeF_3^- in 200 kV Arctica microscope.

a, One representative cryo-EM micrograph of C9orf72:ARF1-SMCR8:WDR41 complex from the 2,579 movies stacks. b, Representative 2D classes. c, Image processing procedure for the dataset collected at 200 kV Talos Arctica.

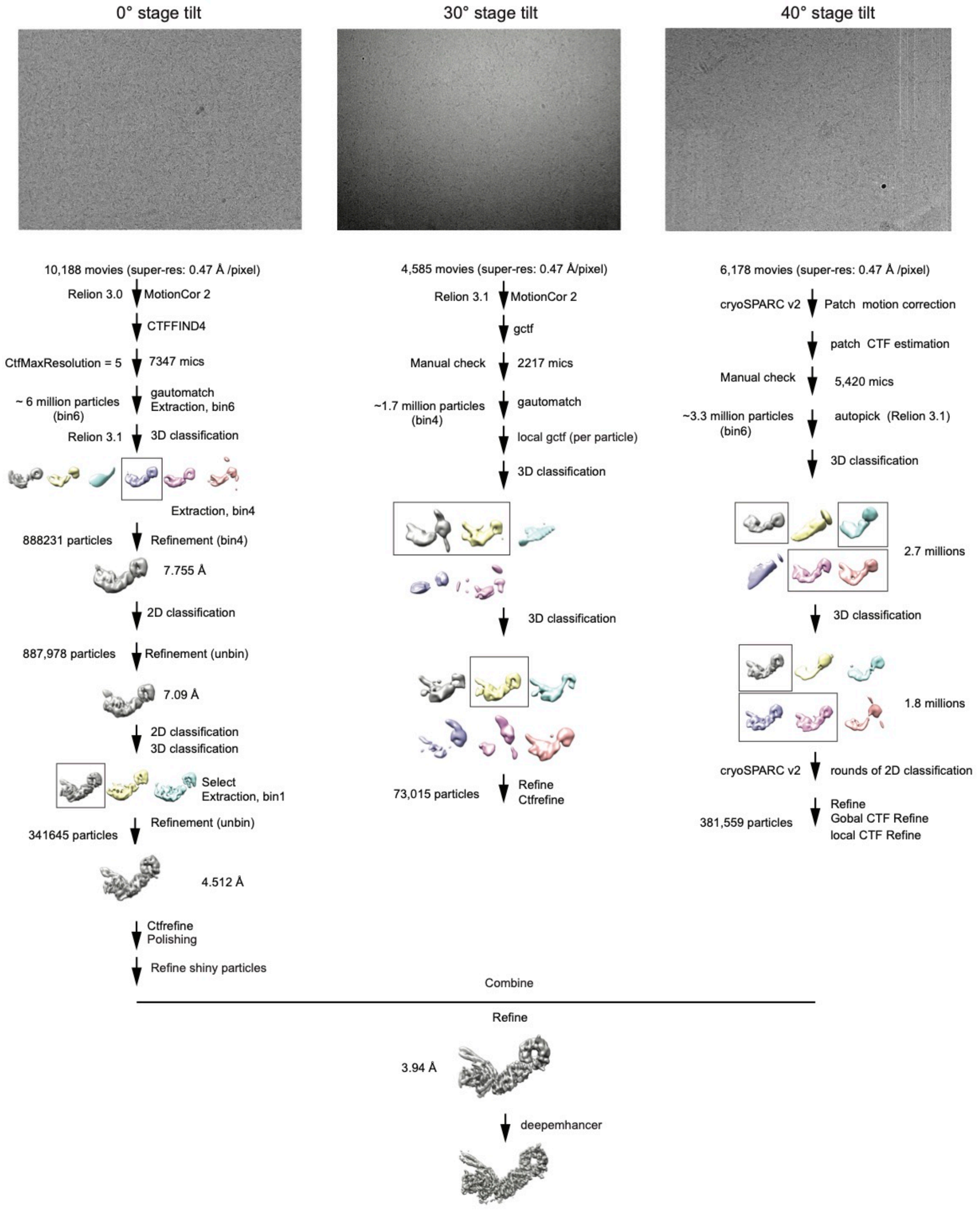

Supplementary Fig 4. Cryo-EM data processing work flow for C9orf72:ARF1-SMCR8:WDR41 complex incubated with BeF_3^- in 300 kV Titan Krios.

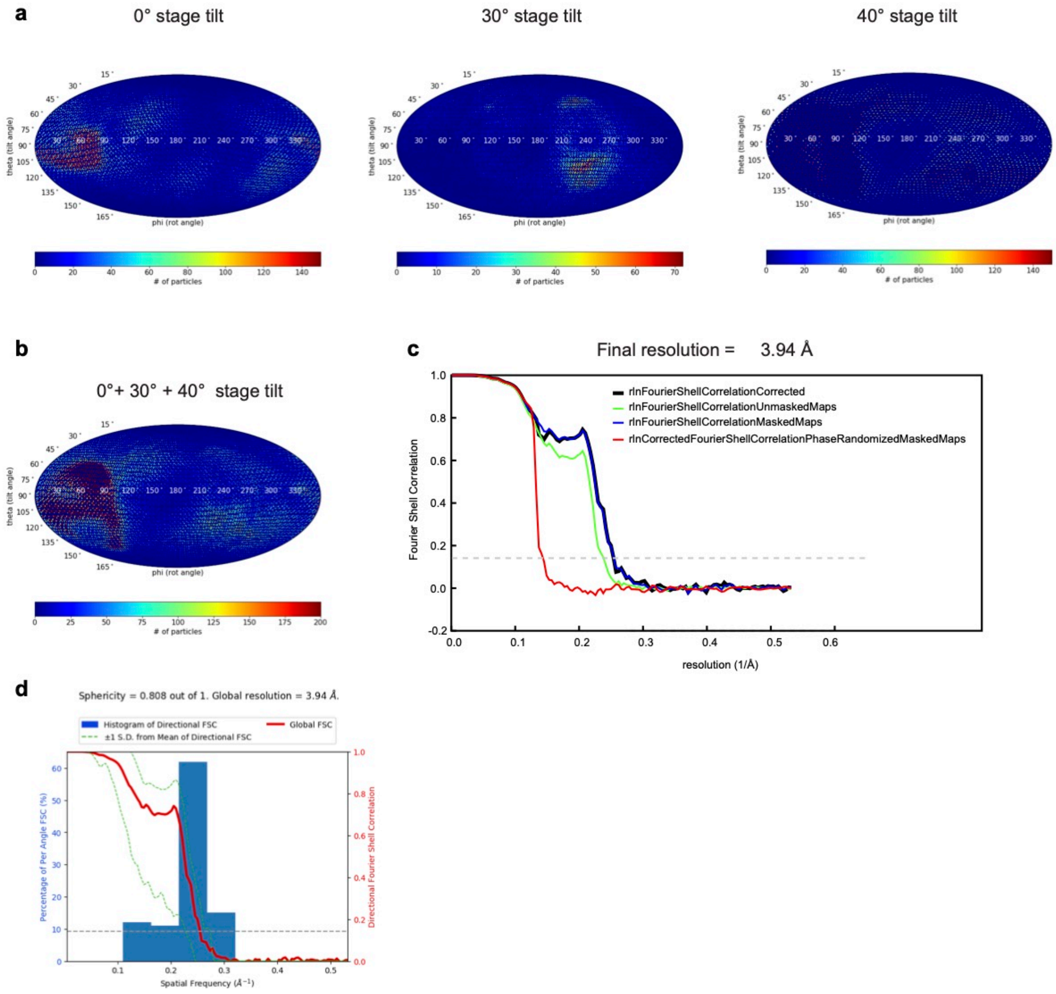

Supplementary Fig 5. Angular distribution of the particles in the reconstructions.

a-b, heatmap of the particle orientations from the 0°, 30° and 40° tilted datasets (a) or combined datasets (b) shown in Mollweide representations. c, Comparison between the FSC curves. d, 3DFSC plot for the final reconstruction.

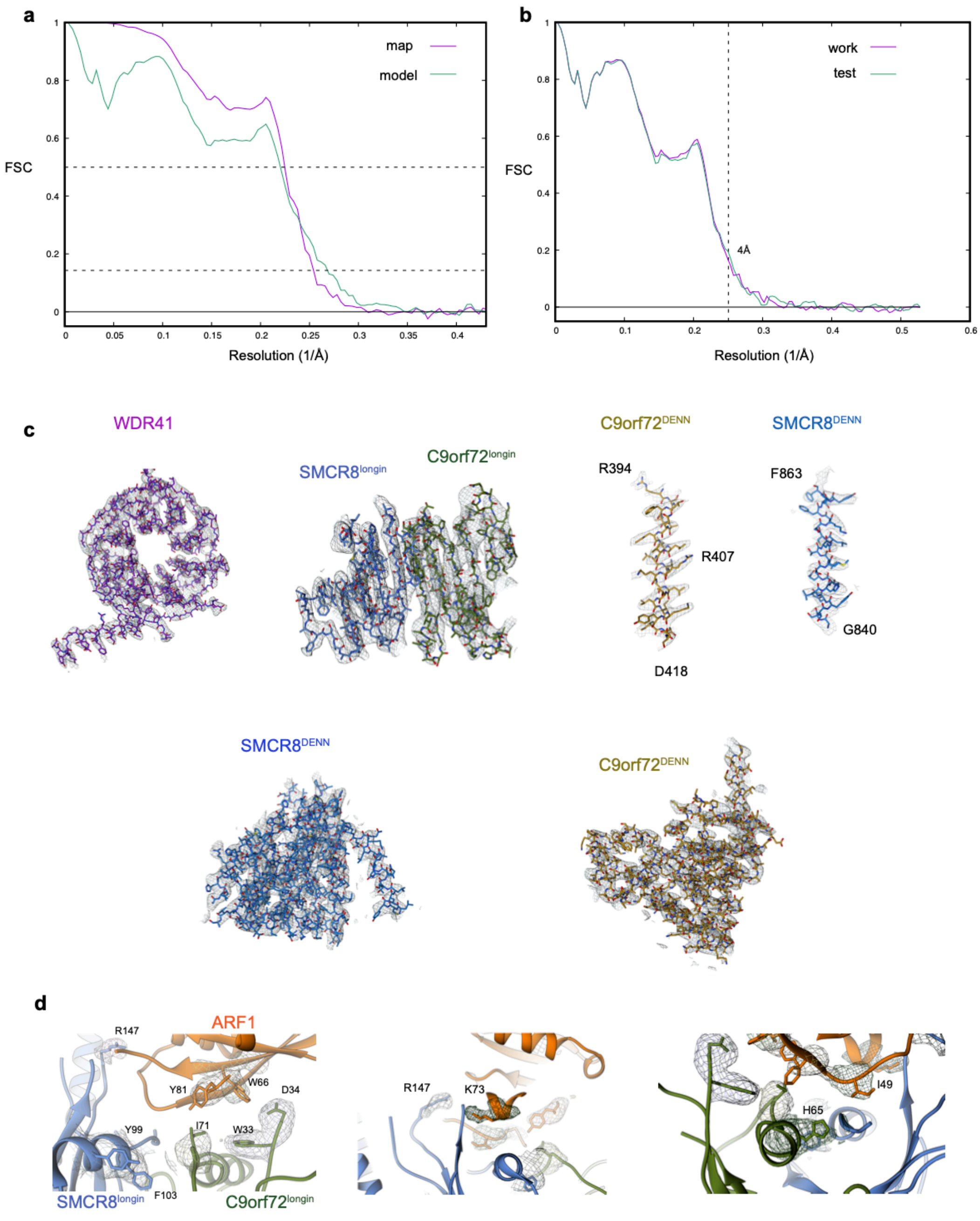

Supplementary Fig 6. Model building and validation.

a, Refinement and map-vs-model FSC. b, Cross-validation test FSC curves to assess over-fitting. The refinement target resolution of 4 Å is indicated c, Refined coordinate model fit of the indicated region in the cryo-EM density. d, The interfacial density between ARF1 and the longin dimers.

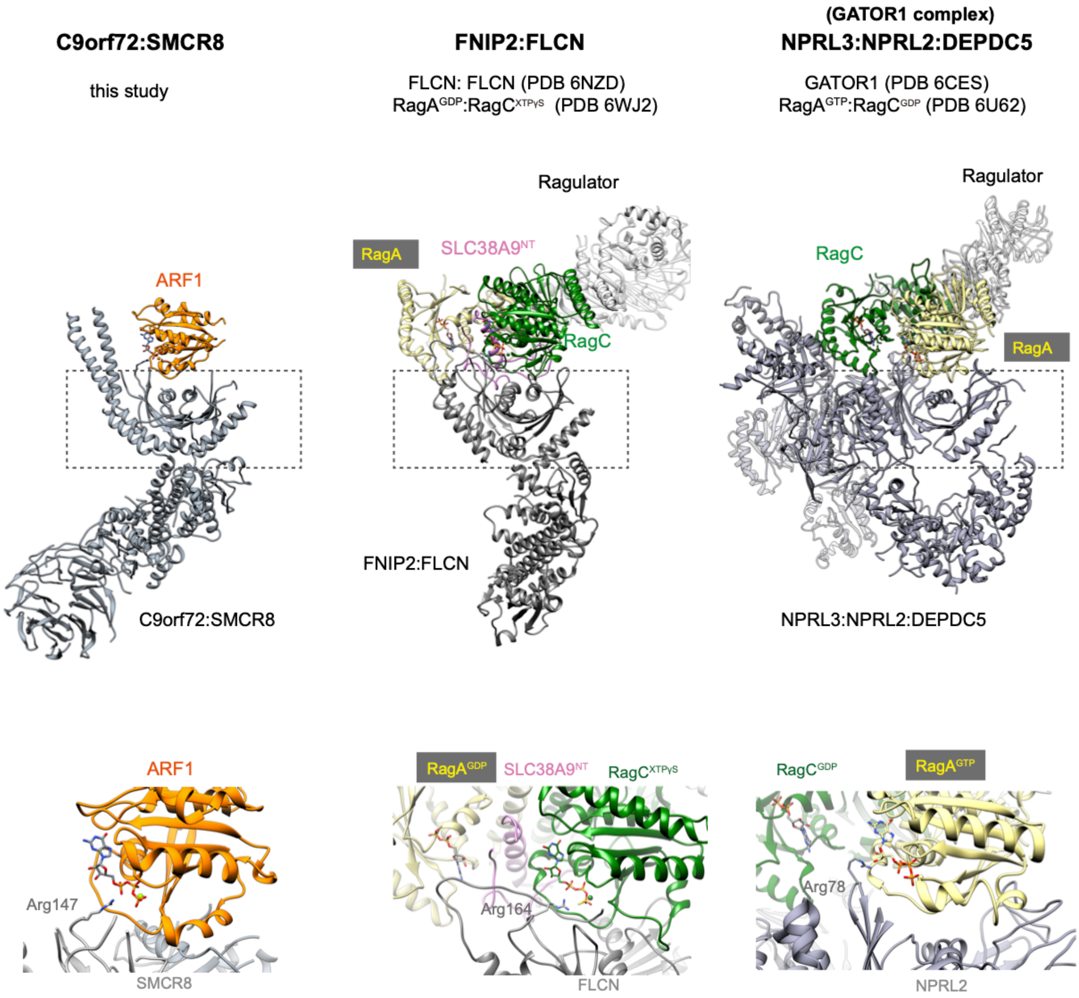

Supplementary Fig 7. The composite models of FLCN:FNIP2 and GATOR1 towards their substrates RagC and RagA, respectively.
The models are generated by aligning the two longin dimers (box with gray lines) from FLCN:FNIP2/GATOR1 to C9orf72:SMCR8 as well as the G domains of either RagC or RagA in their XTP-gammaS or GTP bound state to ARF1.

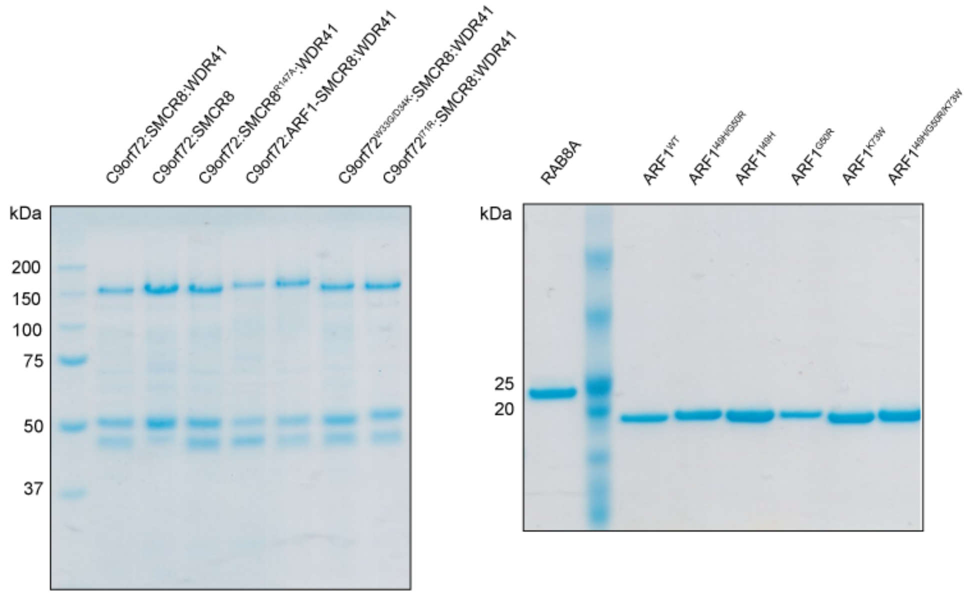

Supplementary Fig 8. SDS-PAGE analysis of the purified proteins used is this study. The 7th lane from the left is a protein sample that is not used in this study, therefore the lane is not labelled. For all the proteins, one representative result from at least two independent experiments is shown.

Uncropped gels

Supplementary Fig 2b

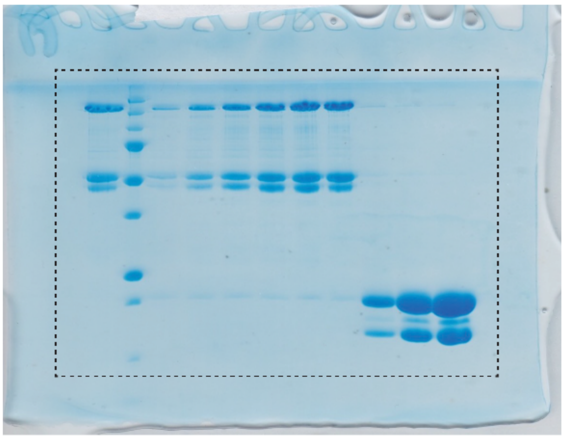

Supplementary Fig 8

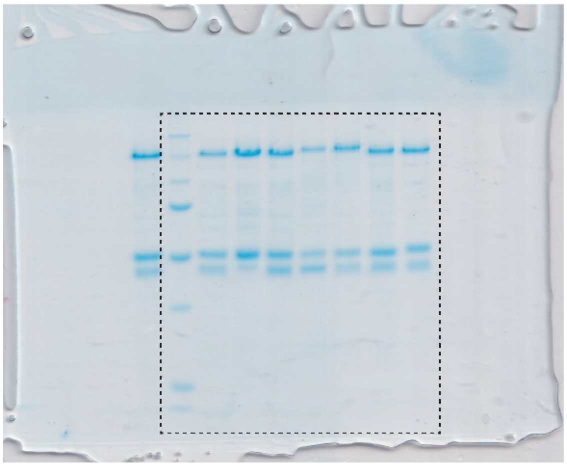

Supplementary Fig 8

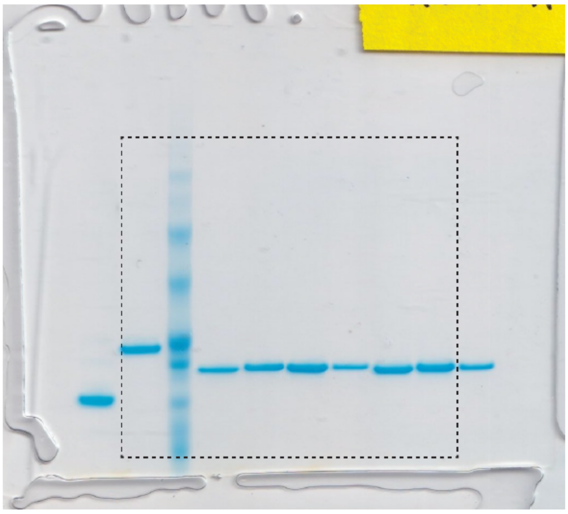

Supplementary Fig 9. Uncropped gels for the Supplementary Figs 2 and 8. The cropping regions are indicated with dash lines.

Supplementary Table 1. Primers used in this study

Primer	Sequence
Kpn1_Arf1_F:	CTGAGGTACCGAAATGCGCATCCTCATG
Arf1_Kpn_R_no stop	TCAGGGTACCCTTCTGGTTCCGGAGCTG
A1M101F:	ACCACCATTCCCACCCACGGCTTCAACGTGGAA
A1M101R	TTCCACGTTGAAGCCGTGGGTGGGAATGGTGGT
A1M102F	ACCATTCCCACCATACGGTTCAACGTGGAAACC
A1M102R	GGTTTCCACGTTGAACCGTATGGTGGGAATGGT
S1M1F	ACCACCATTCCCACCCACCGGTTCAACGTGGAAACC
S1M1R	GGTTTCCACGTTGAACCGGTGGGTGGGAATGGTGGT
A1M5F	GTGGGTGGCCAGGACTGGATCCGGCCCCTGTGG
A1M5R	CCACAGGGGCCGGATCCAGTCCTGGCCACCCAC
Kpn1-8a-F	CTGAGGTACCATGGCCAAAACCTACGACTACC
8a-Xho1-R	CTAGCTCGAGTTAGCTGTTGCCTTCCAGTTTCTTA
A1S8_C9_M1F	GCTACCTTCGCTTACGGCAAGAACATCCTGGGCCCT
A1S8_C9_M1R	AGGGCCCAGGATGTTCTTGCCGTAAGCGAAGGTAGC
A1S8_C9_M2F	ACCCTGAACGGCGAACGCCTGAGAAACGCCGAG
A1S8_C9_M2R	CTCGGCGTTTCTCAGGCGTTCGCCGTTCAGGGT

Supplementary Table 2. Cryo-EM data collection, refinement and validation statistics.

	C9orf72:ARF1-SMCR8:WDR41 (EMD-23827) (PDB 7MGE)
**Data collection and processing**	
Microscope	Titan Krios
Magnification (calibrated)	43,516
Camera	Quantum-K3 Summit
Voltage (kV)	300
Electron exposure (e–/Å^2)	50
Pixel size (Å)	0.94
Symmetry imposed	C1
Initial particle images (no.)	~11 million
Final particle images (no.)	796,219
Map resolution (Å)<br>FSC threshold	3.94<br>0.143
**Refinement**	
Initial model used (PDB code)	1O3Y, 6V4U, 6LT0, 6WHH
Map sharpening *B* factor (Å2)	-
Model composition<br>Non-hydrogen atoms<br>Protein residues<br>Ligands	 9017<br>1323<br>GDP, BeF_3^-, Mg^{2+}
B factor (Å^2, min/max/avg)<br>Protein<br>Nucleotide<br>ligand	 65.50/202.67/122.56<br>146.27/146.27/146.27<br>130.89/140.00/132.71
R.m.s. deviations<br>Bond lengths (Å)<br>Bond angles (°)	 0.003<br>0.687
Validation<br>MolProbity score<br>Clashscore<br>Poor rotamers (%)	 2.16<br>10.98<br>0
Ramachandran plot<br>Favored (%)<br>Allowed (%)<br>Disallowed (%)	 88.04<br>11.48<br>0.48
