# Supplementary material for: Structural basis for the ARF GAP activity and specificity of the C9orf72 complex
Source: Nat Commun. 2021 Jun 18;12:3786. doi: 10.1038/s41467-021-24081-0 (PMC8213707; doi:10.1038/s41467-021-24081-0)
Supplement: Supplementary file 3 — Reporting Summary [file 41467_2021_24081_MOESM3_ESM.pdf]

nature research

Corresponding author(s): James H. Hurley

Last updated by author(s): May 26, 2021

Reporting Summary

Nature Research wishes to improve the reproducibility of the work that we publish. This form provides structure for consistency and transparency in reporting. For further information on Nature Research policies, see our Editorial Policies and the Editorial Policy Checklist.

Statistics

For all statistical analyses, confirm that the following items are present in the figure legend, table legend, main text, or Methods section.

n/a	Confirmed	
☐	☒	The exact sample size (*n*) for each experimental group/condition, given as a discrete number and unit of measurement
☐	☒	A statement on whether measurements were taken from distinct samples or whether the same sample was measured repeatedly
☐	☒	The statistical test(s) used AND whether they are one- or two-sided<br>*Only common tests should be described solely by name; describe more complex techniques in the Methods section.*
☒	☐	A description of all covariates tested
☐	☒	A description of any assumptions or corrections, such as tests of normality and adjustment for multiple comparisons
☐	☒	A full description of the statistical parameters including central tendency (e.g. means) or other basic estimates (e.g. regression coefficient) AND variation (e.g. standard deviation) or associated estimates of uncertainty (e.g. confidence intervals)
☒	☐	For null hypothesis testing, the test statistic (e.g. *F*, *t*, *r*) with confidence intervals, effect sizes, degrees of freedom and *P* value noted<br>*Give P values as exact values whenever suitable.*
☒	☐	For Bayesian analysis, information on the choice of priors and Markov chain Monte Carlo settings
☒	☐	For hierarchical and complex designs, identification of the appropriate level for tests and full reporting of outcomes
☐	☒	Estimates of effect sizes (e.g. Cohen's *d*, Pearson's *r*), indicating how they were calculated

Our web collection on statistics for biologists contains articles on many of the points above.

Software and code

Policy information about availability of computer code

Data collection	SerialEM 3.7.1 (Cryo-EM); Agilent OpenLab CDS (HPLC)
Data analysis	For Cryo-EM data processing, the following software were used: MotionCor2, CTFFIND4.1.13, gctf-v1.06, gautomatch-v0.53, Relion 3.0-beta, cryoSPARC V2, Relion3.1<br>For model building, the following software were used: Coot 0.8.9.1, Phenix 1.19-4092-000 , PyMOL v1.7.2.1, UCSF Chimera 1.13.1 (build 41831), deepemhancer 'tightTarget' and 'highRes' models<br>For data display and analysis, the following software were used: Prism 6 (HPLC result); script from github (https://github.com/fuzikt/starpy) was used to plot for the particle distribution, 3DFSC processing server (https://3dfsc.salk.edu).

For manuscripts utilizing custom algorithms or software that are central to the research but not yet described in published literature, software must be made available to editors and reviewers. We strongly encourage code deposition in a community repository (e.g. GitHub). See the Nature Research guidelines for submitting code & software for further information.

Data

Policy information about availability of data

All manuscripts must include a data availability statement. This statement should provide the following information, where applicable:

- Accession codes, unique identifiers, or web links for publicly available datasets
- A list of figures that have associated raw data
- A description of any restrictions on data availability

EM density map and coordinates have been deposited in the EMDB and rcsb with accession code: PDB ID 7MGE, EMD-23827. These data will be released upon publication of the paper.

Field-specific reporting

Please select the one below that is the best fit for your research. If you are not sure, read the appropriate sections before making your selection.

☒ Life sciences ☐ Behavioural & social sciences ☐ Ecological, evolutionary & environmental sciences

For a reference copy of the document with all sections, see nature.com/documents/nr-reporting-summary-flat.pdf

Life sciences study design

All studies must disclose on these points even when the disclosure is negative.

Sample size	No statistical methods were used to predetermine sample size. The cryoEM data were collected in several sessions which are more than 10,000 movies and manually screened to discard bad micrographs.
Data exclusions	Electron microscopy: micrographs were screened manually and bad ones discarded. After CTF estimation, the bad movies which were bad focus or included ice contamination were excluded.
Replication	GTPases assay were carried out at least triplicates. All replicates were successful and yielded similar results.
Randomization	Randomization is not relevant to this study since no animals or human research participants are involved in this research.
Blinding	Blinding is not relevant to this study since no human or animals are involved in this study.

Reporting for specific materials, systems and methods

We require information from authors about some types of materials, experimental systems and methods used in many studies. Here, indicate whether each material, system or method listed is relevant to your study. If you are not sure if a list item applies to your research, read the appropriate section before selecting a response.

Materials & experimental systems

n/a	Involved in the study
☒	☐ Antibodies
☐	☒ Eukaryotic cell lines
☒	☐ Palaeontology and archaeology
☒	☐ Animals and other organisms
☒	☐ Human research participants
☒	☐ Clinical data
☒	☐ Dual use research of concern

Methods

n/a	Involved in the study
☒	☐ ChIP-seq
☒	☐ Flow cytometry
☒	☐ MRI-based neuroimaging

Eukaryotic cell lines

Policy information about cell lines

Cell line source(s)	HEK 293 Gn-Ti cell lines are purchased from UC Berkeley Cell Culture Facility.
Authentication	The cell lines were authenticated using short tandem repeat analysis.
Mycoplasma contamination	The cell lines were tested negative for mycoplasma contamination using a nuclear stain by fluorescence microscopy.
Commonly misidentified lines (See ICLAC register)	No commonly misidentified cell lines were used.
