# Supplementary material for: Structural basis for the ARF GAP activity and specificity of the C9orf72 complex
Source: Nat Commun. 2021 Jun 18;12:3786. doi: 10.1038/s41467-021-24081-0 (PMC8213707; doi:10.1038/s41467-021-24081-0)
Supplement: Supplementary file 2 — Peer Review File [file 41467_2021_24081_MOESM2_ESM.pdf]

Editorial Note: This manuscript has been previously reviewed at another journal that is not operating a transparent peer review scheme. The manuscript was considered suitable for publication without further review at Nature Communications.
